# Multidrug-Resistant Tuberculosis, Somalia, 2010–2011

**DOI:** 10.3201/eid1903.121287

**Published:** 2013-03

**Authors:** Ireneaus Sindani, Christopher Fitzpatrick, Dennis Falzon, Bashir Suleiman, Peter Arube, Ismail Adam, Samiha Baghdadi, Amal Bassili, Matteo Zignol

**Affiliations:** Author affiliations: World Health Organization, Nairobi, Kenya (I. Sindani);; World Health Organization, Geneva, Switzerland (C. Fitzpatrick, D. Falzon, M. Zignol);; World Health Organization, Hargeisa, Somalia (B. Suleiman);; World Vision International, Nairobi (P. Arube); National TB Control Program, Hargeisa (I. Adam);; World Health Organization, Cairo, Egypt (S. Baghdadi, A. Bassili)

**Keywords:** *Mycobacterium tuberculosis* and other mycobacteria, drug resistance, surveillance, Somalia

## Abstract

In a nationwide survey in 2011, multidrug-resistant tuberculosis (MDR TB) was found in 5.2% and 40.8% of patients with new and previously treated TB, respectively. These levels of drug resistance are among the highest ever documented in Africa and the Middle East. This finding presents a serious challenge for TB control in Somalia.

After ≈2 decades of civil war, Somalia is one of the poorest, least developed, and most violent countries in the world (*1*,*2*). The conflict and violence, particularly in the south-central region, have caused massive population movements, exacerbated recently by severe drought, floods, and famine. In 2011, ≈1.5 million internally displaced persons were reported among the country’s 9 million inhabitants (*2*). Ratios of maternal mortality and deaths of children <5 years of age are among the highest in the world (*1*,*3*), and communicable diseases are the most common causes of illness and death.

In Somalia, tuberculosis (TB) is a serious public health problem.The estimated incidence in 2011 was 300 cases per 100,000 persons, but fewer than half of the estimated cases are actually detected (*4*). Resistance to anti-TB drugs is considered an emerging problem, but the prevalence of multidrug-resistant (MDR) TB (i.e., resistance to rifampin and isoniazid, the 2 most powerful anti-TB drugs) is unknown. To understand the effect of drug-resistant TB and better plan for treatment needs, Somalia’s National Tuberculosis Control Program directly measured drug-resistance prevalence among a representative sample of TB patients in Somalia.

## The Study

During March 2010–October 2011, we conducted a nationwide survey of persons from all 3 zones in the country who had pulmonary sputum smear–positive TB. The survey was designed according to the guidelines of the World Health Organization (WHO) (*5*) by using cluster sampling of 39 diagnostic centers. Patients with sputum smear–negative and extrapulmonary TB were excluded from the study. Cases were classified as newly diagnosed or previously treated according to WHO definitions (*6*). We collected the following variables through a questionnaire administered during sputum collection: patient sex, age, country of birth, and treatment history (new or previously treated). A laboratory in South Africa that is accredited for molecular testing by the National Accreditation System tested sputum samples for resistance to rifampin and isoniazid by using Genotype MTBDR*plus* assay (Hain LifeScience GmbH, Nehren, Germany) (*7**,**8*). The sensitivity of this assay to detect mutations known to confer resistance is higher for rifampin than for isoniazid (98.4% vs. 88.7%, respectively).

The National Ethical Review Board of the Somalia Ministry of Health approved this study. Patients provided informed consent before enrolment. Statistical analyses were performed in Stata (version 12; Stata Corp., College Station, TX, USA). Prevalence estimates were adjusted for fluxes in TB notifications from 2007, the year on which sampling calculations were based, through 2010, the year in which the survey started (Table footnote; Technical Appendix Figure).

**Table:** Prevalence of resistance to first-line antituberculosis drugs in patients with sputum smear–positive pulmonary tuberculosis, Somalia, 2010–2011*

Drug-resistance pattern	Patients, % (95% CI)		
	New, n = 754	Previously treated, n = 96	All
Susceptible	85.6 (81.5–89.7)	46.2 (29.1–63.2)	81.4 (77.7–85.2)
Any resistance to			
Isoniazid	10.9 (7.8–13.9)	51.4 (35.8–67.0)	15.2 (12.2–18.2)
Rifampin	8.7 (5.2–12.1)	43.2 (25.9–60.6)	12.3 (9.1–15.6)
Total	14.4 (10.3–18.5)	53.8 (36.8–70.9)	18.6 (14.8–22.3)
Monoresistance to			
Isoniazid	5.7 (4.1–7.4)	10.6 (0.0–21.5)	6.2 (4.5–7.9)
Rifampin	3.5 (1.1–6.0)	2.4 (0.0–5.4)	3.4 (1.0–5.8)
Total	9.2 (6.1–12.4)	13.0 (1.4–24.5)	9.6 (6.6–12.6)
Multidrug resistance	5.2 (2.8–7.5)	40.8 (24.7–57.0)	8.9 (6.5–11.4)

*Prevalence estimates were obtained by using logistic regression (Stata’s svy: logit command, Stata Corp., College Station, TX, USA) on the binary treatment history variable; each new/retreatment case with a drug-susceptibility test result was weighted by the number of new/retreatment cases notified in its cluster in 2010 (the year in which the survey started), divided by the total number of new/retreated cases with a drug-susceptibility test result in its cluster. The estimation of odds ratios reported elsewhere in the text also included the expansion of categorical sex, age group, and zone variables (*xi: logit*), with clustering and CIs of variance. The findings were robust to multiple imputation of missing data (adding another 78 new and 18 retreatment cases to the sample), use of sampling weights based on 2007 notifications (the year in which cluster samples were calculated), and no use of sampling weights at all; prevalence estimates were equivalent to or slightly higher than those reported here.

A total of 946 patients were consecutively enrolled. Ninety-six enrollees were subsequently excluded because of sample contamination (52 patients), insufficient material to perform the GenoType MTBDR*plus* assay (41 patients), and isolation of *Mycobacteria* spp. other than *M. tuberculosis* (3 patients). The overall total drug susceptibility recovery rate was 89.9%, in line with the country expectations and WHO recommendations (*5*). Of the patients retained in the study (754 persons with new cases and 96 persons with previously treated cases), the male-to-female ratio was 2.4:1.0, and median age was 30 years (range 4–86 years, interquartile range 23–44 years).

MDR TB was detected in 5.2% (95% CI 2.8–7.5) of persons with newly diagnosed TB and 40.8% (95% CI 24.7–57.0) of persons with previously treated TB. Levels of resistance to isoniazid and rifampin and frequencies of any resistance and monoresistance are shown in the Table. The survey detected 87 MDR TB cases.

History of previous anti-TB treatment was the strongest independent factor for MDR TB (odds ratio [OR] 23.0, 95% CI 9.4–56.1, p<0.001), and living in the south-central region or in Puntland was associated with a significantly higher risk for MDR TB than was living in Somaliland (OR 3.6, 95% CI 1.9–6.9 p<0.001 for living in Puntland and OR 4.3, 95% CI 1.7–11.3, p = 0.003 for living in the south-central region). Associations between MDR TB and sex, age, and country of birth were not significant.

## Conclusions

Compared with a study conducted in neighboring Ethiopia in 2005, where MDR TB was found in only 1.6% of new and 11.8% of previously treated TB cases (*9*,*10*), and with surveys conducted in countries of the eastern Mediterranean region, where the average proportion of MDR TB in new and previously treated TB cases was of 3.4% and 29.9%, respectively (*4*), the proportions of MDR TB detected in Somalia are high. At this level of resistance, one would expect that among the 9,760 pulmonary TB cases notified in Somalia in 2011 (*4*), ≈750 were MDR TB and therefore required treatment with second-line drugs; this number does not include other (non-MDR) cases of rifampin-resistant TB that would probably require an MDR TB regimen. This finding presents a real emergency for the National Tuberculosis Control Program considering the duration of second-line treatment (>2 years) (*11*,*12*), the current availability of such treatment in Somalia for a only few patients, and the country’s lack of laboratory capacity to diagnose drug resistance. A systematic review of the cost effectiveness of MDR TB care (*13*) suggests that it would cost US$3,500–5,900 to treat MDR TB on an outpatient basis in Somalia. At <US$400 per disability-adjusted life-year averted, this intervention is cost effective. Efforts are being made to treat MDR TB patients detected in the survey. Because the cost of treating all 750 MDR TB patients is ≈32%–54% of the US$8.2 million (all of it from the Global Fund to Fight AIDS, Tuberculosis and Malaria) that was available to Somalia in 2012 for its entire TB control program (*4*), additional funding will be needed.

In a drug quality survey conducted in Somalia in 2010, 60% of 10 products containing first-line anti-TB drugs that can be easily purchased from pharmacies and informal health care providers met international quality standards (I. Sindani, pers. comm.). The compound most commonly found in insufficient concentration and quality was rifampin. The extensive use of drugs of suboptimal quality, the widespread practice of using wrong medical prescriptions, and incomplete adherence of patients to treatment are the most likely reasons for the high levels of MDR TB in Somalia. These levels appear to be highest in the south-central region, where the security situation is most volatile and disruption of care more frequent. This region also is most affected by recent food shortages (*14*) and has the most internally displaced persons (*15*), factors that are expected to exacerbate disease progression and transmission of *M. tuberculosis*.

This study shows that nationwide surveys to monitor drug-resistant TB are possible even when social conditions are unstable. Two Middle Eastern countries, Afghanistan and Yemen, also were able to conclude nationwide drug resistance surveys in 2011 despite social unrest (*4*). Sample collection, transportation, and monitoring of survey operations in our study have been challenging. The analysis had to account for population movements and changes in the availability of and access to health services.

This study, conducted under difficult circumstances in a country with unstable social conditions, showed that MDR TB is a serious underdetected and widespread public health problem in Somalia. The documented levels of MDR TB are among the highest reported in Africa and the Middle East and suggest that ≈750 patients in the country had MDR TB in 2011. Urgent measures should be introduced to improve access to diagnosis of drug resistance and availability of second-line medication for all patients who need them.

Technical Appendix Figure Percentage change in total tuberculosis notifications by diagnostic center participating in the survey, Somalia, 2007–2010.
