# Correction: Vol. 19, No. 3

**DOI:** 10.3201/eid2011.C12011

**Published:** 2014-11

**Keywords:** errata, erratum, correction

The affiliations of author Amal Bassili were listed incorrectly in the article Multidrug-Resistant Tuberculosis, Somalia, 2010–2011 (I. Sindani et al.). She was affiliated with the World Health Organization, Cairo, Egypt, and the Medical Research Institute, Alexandria University, Alexandria, Egypt. The article has been corrected online (http://wwwnc.cdc.gov/eid/article/19/3/12-1287.htm). 

