# Supplementary material for: Multidrug-Resistant Tuberculosis, Somalia, 2010–2011
Source: Emerg Infect Dis. 2013 Mar;19(3):478–80. doi: 10.3201/eid1903.121287 (PMC3647667; doi:10.3201/eid1903.121287)
Supplement: Technical Appendix Figure — Percentage change in total tuberculosis notifications by diagnostic center participating in the survey, Somalia, 2007–2010. [file 12-1287-Techapp-s1.pdf]

Publisher: CDC; Journal: Emerging Infectious Diseases
Article Type: Dispatch; Volume: 19; Issue: 3; Year: 2013; Article ID: 12-1287
DOI: 10.3201/eid1903.121287; TOC Head: Dispatch
Article DOI: http://dx.doi.org/10.3201/eid1903.121287

Multidrug-resistant Tuberculosis, Somalia, 2010–2011

Technical Appendix

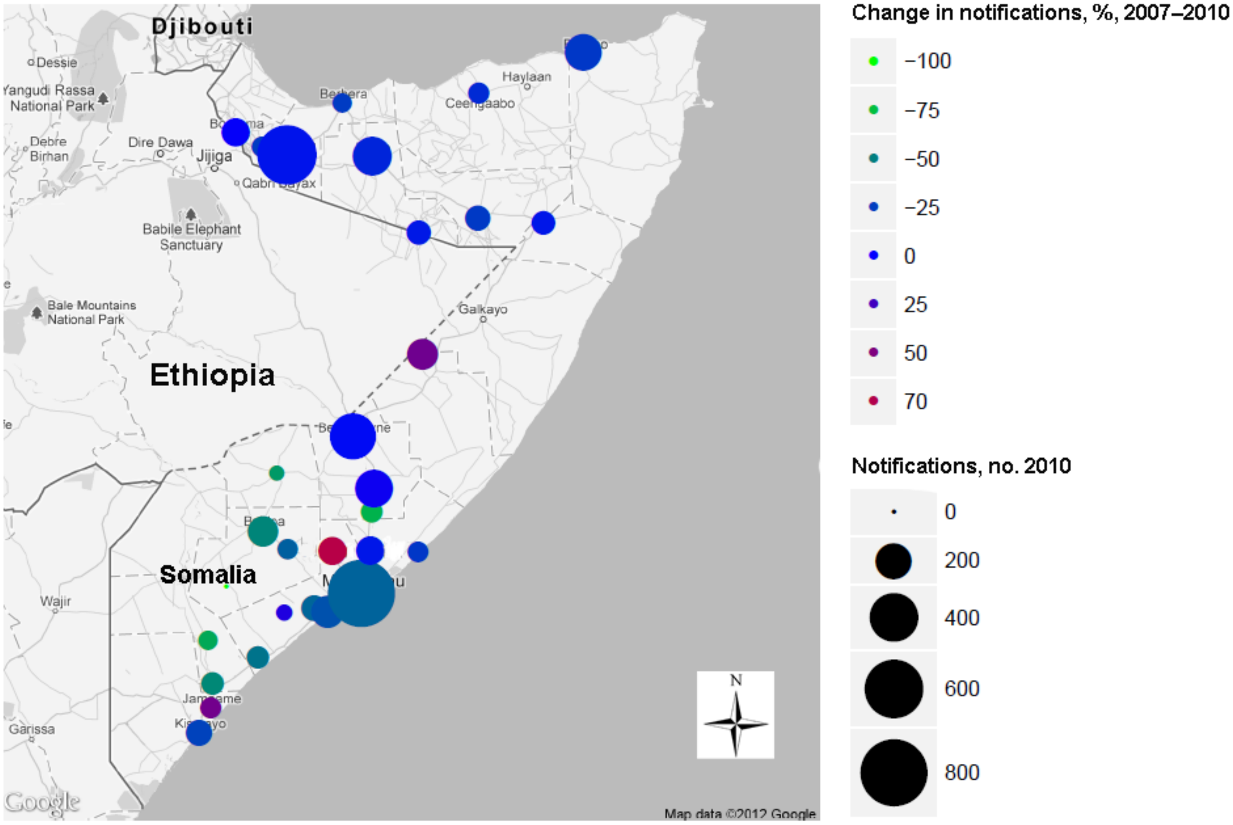

Technical Appendix Figure. Percentage change in total tuberculosis notifications, by diagnostic center participating in the survey, Somalia, 2007–2010.
